# Addressing Challenges in Obtaining Emergency Medicine Away Rotations and Standardized Letters of Evaluation Due to COVID-19 Pandemic

**DOI:** 10.5811/westjem.2020.3.47444

**Published:** 2020-04-02

**Authors:** Linda Katirji, Liza Smith, Alexis Pelletier-Bui, Emily Hillman, Xiao Chi Zhang, Michael Pasirstein, Mark Olaf, Jazmyn Shaw, Douglas Franzen, Ronnie Ren

**Affiliations:** * University of Kentucky, Department of Emergency Medicine, Lexington, Kentucky; † University of Massachusetts Medical School, Baystate Medical Center, Department of Emergency Medicine, Springfield, Massachusetts; ‡ Cooper Medical School of Rowan University, Department of Emergency Medicine, Camden, New Jersey; § University of Missouri—Kansas City School of Medicine, Truman Medical Center, Department of Emergency Medicine, Kansas City, Missouri; ¶ Thomas Jefferson University, Department of Emergency Medicine, Philadelphia, Pennsylvania; || Drexel University College of Medicine, Department of Emergency Medicine, Philadelphia, Pennsylvania; # Geisinger Commonwealth School of Medicine, Department of Emergency Medicine, Scranton, Pennsylvania; ** University of Mississippi School of Medicine, Univeristy of Mississippi Medical Center, Jackson, Mississippi; †† University of Washington, Department of Emergency Medicine, Seattle, Washington

With the unpredictable future of the coronavirus disease 2019 (COVID-19) pandemic, institutions have begun altering the clinical experience for students and instituting travel bans for both their faculty and students.^1^ On March 17, 2020, a joint recommendation from the Association of American Medical Colleges and the Liaison Committee on Medical Education was issued, which supported suspending clinical activities for medical students for a two-week minimum.^2^ There exists precedence for sudden medical school curricular adaptations in response to emerging diseases and disasters, including alterations in planned didactics, distance learning, and other methods of risk mitigation.^3^–^5^ Numerous institutions have begun canceling clerkship rotations for visiting students, while others are prohibiting their own students from traveling to complete away rotations. While many institutions have initiated video conferencing and virtual simulation in lieu of clinical exposure, there is increasing concern that these students will suffer from limited opportunities to evaluate and treat patients in emergency department settings and to receive real-time assessment of their clinical skills.

The Council of Residency Directors in Emergency Medicine (CORD) Advising Students Committee in Emergency Medicine (ASC-EM) anticipates institutional and regional variability in both the spread and response to COVID-19. Travel restrictions and host institution rotation closures will impact the number of emergency medicine (EM) rotations EM-bound medical students can complete in an unprecedented manner. They may prevent students from completing any away rotations this academic cycle, challenging the students’ collective ability to obtain EM Standardized Letters of Evaluation (SLOE) outside of their home institution. For students without a home EM program to rotate in, they may not have the ability to obtain any SLOE at all, which could be devastating to their EM residency application.^6^,^7^

Historically, SLOEs obtained from home and away EM rotations have served as important tools to determine which candidates to invite for residency interviews.^6^–^9^ Approximately 80% of EM programs will not consider an applicant for an interview unless they have at least one SLOE.^10^,^11^ In the most recent review of the Emergency Medicine Residents’ Association (EMRA) Match website, of the 175 out of 258 programs self-reporting a SLOE requirement, only 13 programs (7%) stated they would review an applicant without a SLOE while 79 programs (45%) stated they required two SLOEs before considering an applicant for interview.^12^

Over the past decade, the group SLOE, written by the EM clerkship and residency education team at a residency program, has become the preferred SLOE for residency EM applications.^6^,^8^,^13^,^14^ Program directors have since held single-author SLOEs, SLOEs from EM faculty not affiliated with a residency program, and non-SLOE format letters of recommendation (LoR) in lesser regard.^6^,^15^ In the present COVID-19 pandemic, students without a residency-based home EM rotation will face markedly greater barriers to obtaining these coveted group SLOEs. EM’s emphasis on residency group SLOEs over other letter types creates an undue burden on these vulnerable students and makes the application process intrinsically inequitable. This inequity warrants a reevaluation of the current application practice.

In this continuously evolving, exceptionally challenging time, it is important for the educational community to face these challenges in a united front. ASC-EM proposes the following recommendations for all stakeholders, including EM program leadership, medical schools, and EM-bound medical students, to consider for the upcoming EM application cycle.

**For Program Directors:**

1. Programs should be flexible with their SLOE requirements.
  - ASC-EM recommends for application cycle 2020–2021 that residency program leadership consider reducing the number of SLOEs needed to review an application to **one SLOE (or fewer)** to account for students who cannot obtain a SLOE at their home institution or from away rotations. We also recommend programs to accept alternative letters of recommendations to act as surrogates for their typical group SLOE requirements as detailed in the paragraphs below.
2. Programs should give weight to alternative LoRs that include the traditional SLOE content.
  - Examples of alternative LoRs include, but are not limited to, a SLOE from a home EM rotation at an institution without an associated EM residency program (“orphan” SLOEs),EM sub-specialty SLOEs (ultrasound, toxicology, pediatric EM, emergency medical services, or other sub-specialties), and LoRs written by advisors for the instance that a student has been entirely unsuccessful in obtaining an EM rotation. The CORD website contains instructions and a template for writing such SLOEs.^16^
  - Given the increased emphasis on alternative LoRs, letter writers must be instructed to address relevant clinical and professional competencies typically seen in the “Qualifications for EM” section of SLOEs. A standard template for this can be found on the CORD website.^17^

**For Deans and Letter Writers:**

3. Writers should use clear language to reflect a student’s loss of opportunities.
  - Medical Student Performance Evaluation (MSPE): Due to the anticipated institutional and regional variability, ASC-EM recommends that institutions include a clear, explicit statement in their MSPE explaining any institutional policy limiting their students’ ability to complete EM rotations.
  - SLOE and alternative LoRs: ASC-EM recommends the inclusion of the following standard verbiage in SLOEs and alternative LoRs to identify students that could not obtain the recommended number of rotations:
    “This student was unable to obtain the expected number of residency SLOE opportunities due to uncontrollable circumstances surrounding the COVID-19 pandemic. These circumstances include [include all that apply] home institution prohibiting school-related travel, cancellation of home EM rotation, cancellation of EM away rotations the student had accepted, and inability to find an EM rotation willing to accept students.”

**For Medical Students:**

4. Students should consider going on fewer away rotations.
  - We anticipate EM rotations that accept visiting students will become a scarce opportunity that must be shared to maintain a healthy application environment. We ask all stakeholders in the EM application process, including but not limited to faculty advisers, clerkships, and students, to be cognizant of the number of EM rotations each student chooses to complete. Given the possibility of drastically limited EM rotation positions, ASC-EM would like to revise the number of away rotations we have recommended students complete in previous application cycles.
  - Students who can rotate at their home EM program: In the event that a student is able to both travel to institutions accepting visiting students and secure available clerkship positions, that student should not complete more than one away rotation.
  - Students without a home EM program: Students should not complete more than two away rotations.

**For Institutions and Clerkship Directors Still Accepting Visiting Students:**

5. Clerkship directors and medical schools should preferentially consider students without a home EM program for an EM clerkship at their institution.
  - To yield a more equitable distribution of scarce audition rotation opportunities, host institutions and their clerkship directors should actively seek applicants who are unable to obtain a SLOE from their home institution.
  - Applicants without a home EM rotation, whether it be due to restrictions on students rotating in their home EM department or lack of a home EM residency program altogether, should communicate that status in their visiting clerkship application, if possible.
  - CORD ASC-EM maintains a living document of medical schools where students lack access to a home EM program, so-called orphan programs. Application reviewers should use this tool to aid their decision making when determining which students to invite for visiting opportunities. The most up to date document can be found here.

**For Stakeholders Involved in the Restriction of Visiting Rotations:**

6. The status of EM rotations should be clear and accurate.
  - The status of visiting EM rotations should be accurately represented on relevant platforms such as Visiting Students Learning Opportunities (VSLO). Institutions should work with their respective application platforms to ensure that students cannot apply to rotations that have been or will be canceled due to the COVID-19 pandemic. We encourage institutions to keep the availability indicator for their rotation on EMRA Match for Clerkships up to date as well as the “Information Students Should Know” section for relevant COVID-19 updates.^12^ Students should not be expected to apply to rotations uncertain of whether the rotation is open or canceled.
7. Students should be protected from financial implications of canceled rotations.
  - Financially, students are the most vulnerable group among all stakeholders. Institutions restricting their students from traveling should help their students recoup the money already spent on setting up visiting rotations. Host institutions canceling their visiting rotations should work with their application platform, such as VSLO, to refund students who have already applied to or accepted a rotation there.

We understand that these proposed changes may be uncomfortable for programs that have relied on SLOEs to be the most important representation of a student’s abilities, and for students who are eager to be able to demonstrate their competencies in the audition setting.^6^–^8^ Ultimately, these recommendations are motivated by the need to preserve the health and safety of our EM community and to ensure that students who traditionally are at the greatest disadvantage in navigating the application process are not excluded entirely from consideration.

Although the COVID-19 pandemic was the impetus for these unique recommendations, our response may be applicable to future, unforeseen circumstances that alter the usual application process. Emergency physicians are known to be adaptable to their ever-changing clinical environment, and we are confident that our flexibility will extend to the academic realm as well.
